# IFNγ regulates ferroptosis in KFs by inhibiting the expression of SPOCD1 through DNMT3A

**DOI:** 10.1038/s41420-024-02257-z

**Published:** 2025-01-16

**Authors:** Xiuxia Wang, Yating Yang, Xianyu Zhou, Shun Yu, Xusong Luo, Lin Lu, Zhen Gao, Jun Yang

**Affiliations:** 1 Department of Plastic and Reconstructive Surgery, Shanghai Ninth People’s Hospital, Shanghai Jiaotong University School of Medicine, Shanghai, 200011 China; 2 The Affiliated Hospital of Jiangnan University, Jiangsu, China

**Keywords:** Necroptosis, DNA metabolism

## Abstract

Keloid is benign skin tumor, and their curing is relatively difficult due to the unclear mechanism of formation. Inducing ferroptosis of keloid fibroblasts (KFs) may become a new method for treating keloid. Here, we discover interferon (IFN)γ could induce KFs ferroptosis through inhibiting SPOC domain-containing protein 1 (SPOCD1), serving as a mode of action for CD8^+^T cell (CTL)-mediated keloid killing. Mechanistically, keloid IFNγ deficiency in combination with reduced DNMT3A increase the expression of SPOCD1, thereby promoting KFs’ proliferation and inhibiting its ferroptosis. Moreover, keloid SPOCD1 deficiency attenuates KFs progression and extracellular matrix (ECM) deposition. Reducing IFNγ and SPOCD1 simultaneously can increase the positive rate of reactive oxygen species (ROS) and promote mitochondrial shrinkage. Ex-vivo explant keloid culture has also confirmed that the reduction of SPOCD1 helps to reduce the proliferation rate of KFs, inhibit the angiogenesis of keloid scars, and thus inhibit keloid formation. Thus, IFNγ signaling paired with SPOCD1 is a natural keloid ferroptosis promoting mechanism and a mode of action of CTLs. Targeting SPOCD1 pathway is a potential anti-keloid approach.

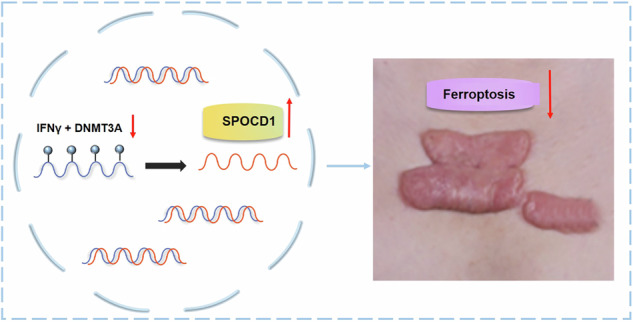

## Introduction

Keloid arises from hyperactive fibroblasts producing excessive collagen and growth factors, stemming from aberrant wound healing in response to skin trauma or inflammation [1]. Clinically, it presents as a persistent skin lump infiltrating and expanding into adjacent healthy tissues, accompanied by localized itching and discomfort, significantly impacting the patient’s physical and psychological well-being [2]. Notably, keloid shares several biological characteristics with tumors, including heightened invasiveness and recurrence propensity [3, 4]. The recurrence rate of keloid following conventional surgical intervention approaches nearly 100%. While a combination of surgery and radiation therapy can mitigate recurrence to approximately 10%, concerns over the carcinogenic potential of radiation therapy often deter patients. Moreover, alternative treatments like drug therapy and laser therapy still yield high recurrence rates [5]. Consequently, preventing keloid recurrence post-treatment remains a formidable global challenge within plastic surgery and the broader surgical domain.

Keloid fibroblasts (KFs) play a pivotal role in the genesis and progression of keloid. Compared to their normal fibroblasts (NFs), KFs exhibit heightened abilities in proliferation, migration, invasion, and extracellular matrix (ECM) production. Scientists widely acknowledge that transforming growth factor-β1 (TGF-β1) plays a central role in fostering KFs growth [6]. Our prior investigations revealed the following: 1) While employing a TGF-β peptide antagonist effectively curbs hypertrophic scar fibroblast growth, its efficacy against KFs remains limited; 2) Utilization of adipose stem cell conditioned culture medium demonstrates inhibition of TGF-β1 expression in KFs, consequently impeding collagen 1 (col1) synthesis, a crucial ECM component, yet it fails to suppress col3 expression [7, 8]. Additionally, Zhao Tao et al. delineated various approaches targeting TGF-β1 signaling pathway for keloid treatment, albeit without achieving a definitive cure [9]. This underscores the intricate nature of keloid formation and progression, suggesting that solely inhibiting TGF-β1 might not suffice for keloid remission. Hence, could promoting KFs apoptosis offer a more effective approach to keloid treatment?

Cell growth and death are inherently interconnected processes that coexist in biological systems. Literature reports highlight the pivotal role of ferroptosis in inducing tumor cell death and combating tumors [10]. The presence of ferroptosis in tumor cells suggests a promising avenue for leveraging this phenomenon to impede KFs growth and ultimately address keloid. Building upon this premise, our further investigation into KFs ferroptosis underscores its potential for keloid treatment by instigating ferroptosis in KFs. Consequently, delving into the molecular mechanisms underlying ferroptosis in KFs and identifying critical targets to thwart the occurrence and progression of keloid hold significant guiding implications for clinical interventions.

Through RNA sequencing (RNA-seq), we discovered that SPOCD1, which plays a regulatory role in some kinds of cells’ death [11, 12], is overexpressed in keloid. In a study examining the alterations in ferroptosis-related genes in patients with glioblastoma, SPOCD1 emerged as a high-risk gene [13]. This suggests that SPOCD1 is likely involved in cell ferroptosis, which is precisely the focus of our ongoing research about keloid.

## Results

### SPOCD1 is highly expressed in keloids

**Fig. 1:**
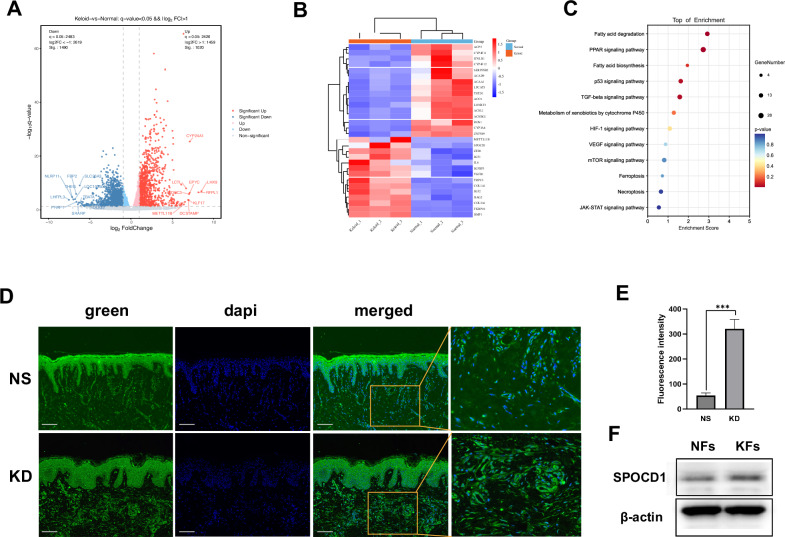
SPOCD1 is highly expressed in keloids. **A**, **B** The volcano and heatmap plot illustrate the differentially expressed genes related to fibroblasts’ activities of the keloid group compared to the control group. **C** The GO function enrichment analysis of these differentially expressed genes. **D**, **E** Immunofluorescence staining and quantification of SPOCD1 in KD and NS (control group). **F** Western blotting assay of SPOCD1 in KFs and NFs. Bar=100 μm. *p < 0.05, **p < 0.01, ***p < 0.001.

To investigate the disparities between keloids and normal skin, RNA-seq was utilized to analyze the activity of various cells involved in keloid. The heatmap and Volcano Plot analysis identified 2510 differentially expressed genes (DEGs) including SPOCD1 between keloid and normal skin tissue (Fig. 1A, B). Genes significantly upregulated in the keloid group were primarily associated with ferroptosis, extracellular matrix structural constituent, wound healing and cellular response to transforming growth factor beta stimulus. Gene Ontology (GO) enrichment analysis highlighted those biological processes such as iron ion binding, long-chain fatty acid metabolic process, and iron ion transport were all upregulated in the keloid group compared to the Control group (Fig. 1C).

Followed by SPOCD1 was discovered significantly expressed in keloid, cellular distribution and quantitative analysis of SPOCD1was detected with either immunofluorescence and western blotting (Fig. 1D–F). The intensity of fluorescence staining also show that SPOCD1 is highly expressed in keloid as fluorescence intensity of keloid is 2.3 times higher than that of normal skin and so as the western blotting results (Fig. 1E, F).

### SPOCD1 could regulate the ferroptosis of KFs

The above RNA-seq analysis has found that the expression of SPOCD1 is significantly increased in typical keloid tissue samples. So, what role does SPOCD1 play in keloid or KFs?

**Fig. 2:**
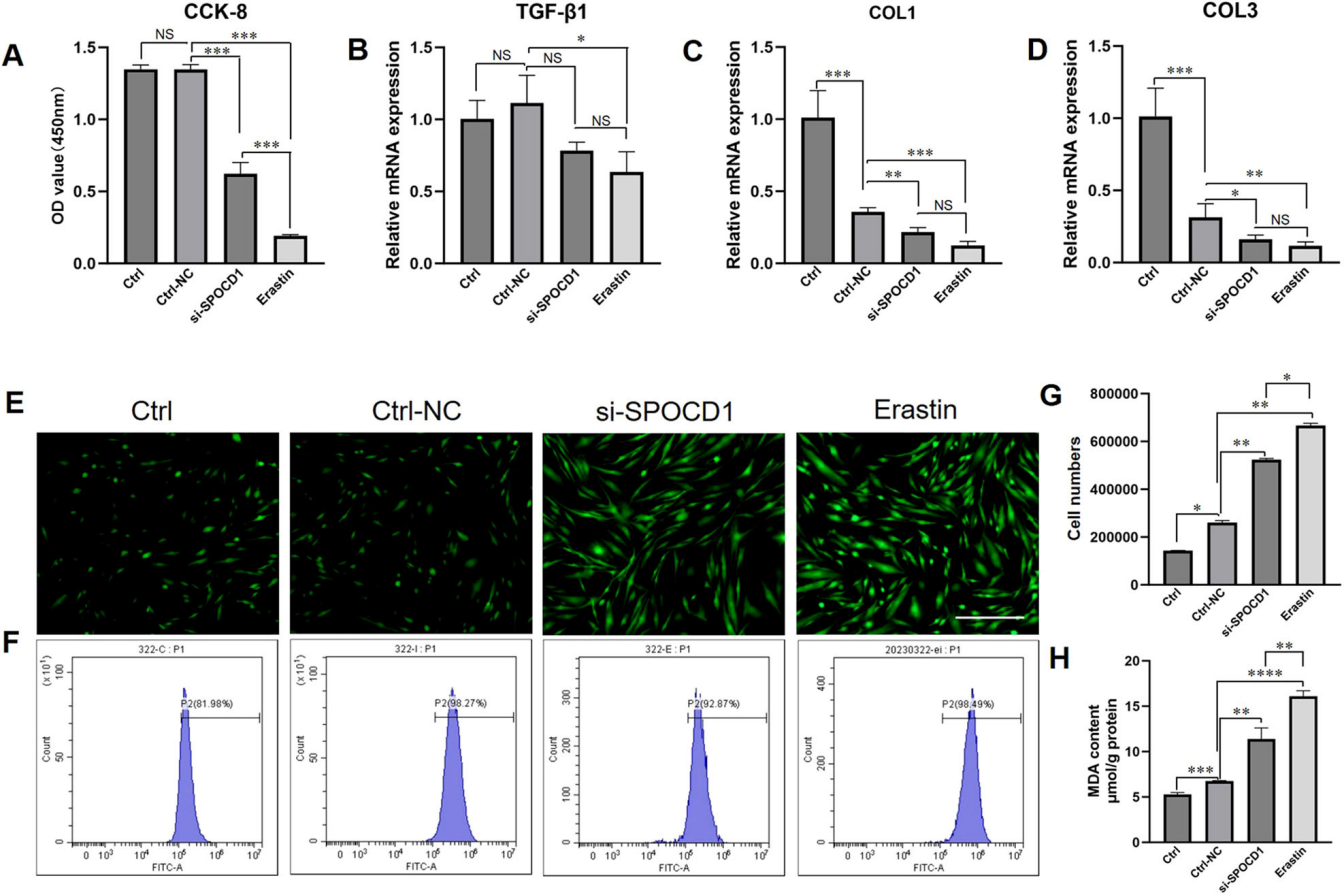
SPOCD1 could regulate the ferroptosis of KFs. **A** the cck-8 assay of KFs after treated with si-SPOCD1 and erastin(5 μM) for 72 h. **B**–**D** the gene expression of TGF-β1, col1 and col3 of KFs after treated with si-SPOCD1 and erastin (5 μM) for 24 h. **E** Fluorescence intensity detection of ROS of KFs after treated with si-SPOCD1 and erastin(5 μM) for 24 h. **F** Flow analysis of ROS positive cells of KFs after treated with si-SPOCD1 and erastin (5 μM) for 24 h. **G** Quantitative statistics of ROS positive cells. **H** Quantitative statistics of MDA. Bar = 100 μm.*p < 0.05, **p < 0.01, ***p < 0.001.

We down-regulated the expression of SPOCD1 using small interfering RNA (si-RNA) technology (Suppl. table S3). After three days of cultivation, we observed that although the cell proliferation ability of the si-SPOCD1 group was higher than that of the erastin (a ferroptosis inducer) group, it was significantly lower than that of the control or Ctrl-NC group for 72.26 ± 3.47% (Fig. 2A). We then utilized RT-QPCR technology to examine the TGF-β1, which promotes the growth of KFs. We discovered that the biological function of SPOCD1 has no links with TGF-β1(Fig. 2B). Specifically, si-SPOCD1 cannot decrease or increase the gene expression of TGF-β1. We also assessed the main matrix components col1 and col3 in KFs, and observed that si-SPOCD1 can reduce their expression by over 50%, yet there is no statistically significant difference compared to the erastin group (Fig. 2C, D).

Given that SPOCD1 can promote the growth of KFs and exhibits an inverse relationship with erastin, might SPOCD1 be involved in regulating the ferroptosis activity of KFs? As Iron serves as a cofactor for a host of biochemical processes, including oxygen storage, oxidative phosphorylation, and enzymatic reactions required for cellular proliferation, it is essential for the survival of nearly all organisms [14]. However, the levels of free iron in a cell must be tightly regulated to avoid the generation of reactive oxygen species (ROS) via the Fenton reaction which will lead to cell death [15, 16]. Hence, ROS accumulation has been one of the hallmarks of ferroptosis. We, thus, applied DCFH-DA (2′, 7′-dichlorodihydrofluorescein diacetate), a cell-permeable fluorescent probe, to detect intracellular ROS in KFs.

After incubation with si-SPOCD1, erastin and or both, DCFH-DA was added to the dish and reacted with cells for1h. The confocal imaging revealed that SPOCD1 not only participates in the regulation of ferroptosis in KFs, but also effectively reduces ferroptosis in KFs. This is not only reflected in the higher fluorescence intensity of the si-SPOCD1 group compared to the control group (Fig. 2E), but also in the flow cytometry results (Fig. 2F), as the number of ROS positive cells in si-SPOCD1 group shows a significant increase(Fig. 2G) and so as the MDA results(Fig. 2H). Although there is a significant difference between the si-SPOCD1 group and the positive control group (erastin group), it is significantly smaller than the percentage between the erastin group and the control group. The results of GSSG/GSH once again confirmed our findings(Suppl. Figs.4).

### keloid are impacted by DNA methylation and exhibits hypomethylation

**Fig. 3:**
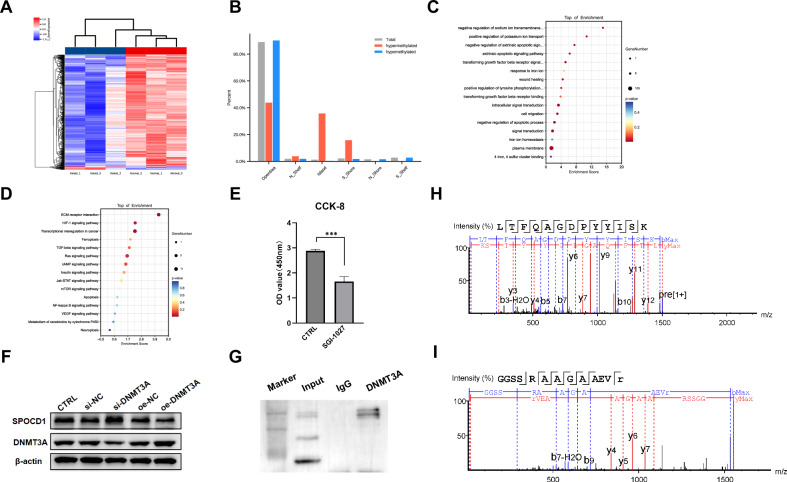
Keloid are impacted by DNA methylation and so as the SPOCD1. **A** Heatmap of hierarchical clustering analysis for samples according to DMPs. **B** The distribution of keloid’s DNA methylation sites. **C** The GO function enrichment analysis of these differentially expressed genes regulated by DNA methylation, showing the top 16 enrichment results. **D** KEGG enrichment results for significantly expressed genes with altered DNA methylation in keloid, showing the top 15 enrichment results. **E** The CCK-8 assay of KFs after treated with SGI-1027(5 μM) for 5 h. **F** The expression of protein SPOCD1 and DNMT3A after treated with si-DNMT3A or oe-DNMT3A for 72 h. **G** Western blotting results of DNMT3A in KFs. **H** Ion strength of protein mass spectrometry analysis. **I** The ion strength of SPOCD1 as determined by mass spectrometry analysis. *p < 0.05, **p < 0.01, ***p < 0.001.

According to literature reports, SPOCD1 might contribute to the epigenetic regulation of gene expression in sperm cells by influencing DNA methylation, histone modifications, or other epigenetic marks that affect chromatin structure and function [17, 18]. Therefore, we speculate that the regulatory activity of SPOCD1 in keloid is also related to DNA methylation. Initially, bisulfite-converted DNA underwent analysis on an Illumina Infinium Methylation EPIC 850 K Bead Chip (Illumina). The results from genome-wide DNA methylation analysis of three keloid tissues exhibited significantly lower DNA methylation levels at multiple loci/genes compared to three normal skin samples corresponding to keloid. The gene regions where the probes are located on the Methylation EPIC chip are categorized into IGR, TSS1500, TSS200, 5’UTR, 1stExon, Body, ExonBnd, and 3’UTR. Likewise, the CpG island region where the probes are situated is divided into N shelf, N shore, CpG Island, S shore, and S Shelf. Employing a threshold of |Log2 fold change | > 0.1 and *P*-value < 0.05, we visualized differentially methylated regions (DMRs) between keloid and normal skin (*n* = 3 per group) using the “pheatmap” R package. In total, 913 DMRs were identified, with 888 DMRs showing decreased methylation in keloid tissue. These DMRs are arranged based on their β-values and represented as a heatmap in Fig. 3A.

The GO database offers specialized terminology to delineate the attributes of gene products. It encompasses three principal categories: Biological Process (BP), which characterizes molecular activity events and functional cell sets, tissues, organs, and species, often closely linked to experimental research inquiries; Cellular Component (CC), which identifies the cell or its extracellular milieu; Molecular Function (MF), defining the active elements describing gene products at the molecular level (Fig. 3C). The Kyoto Encyclopedia of Genes and Genomes (KEGG) is a database systematically analyzing gene function and establishing connections between genomic and functional information. In our study, we conducted separate analyses using GO and KEGG and found that DNA methylation significantly influences the regulation of aberrant keloid hyperplasia and ferroptosis (Fig. 3D).

Next, we introduced SGI-1027 (5 μM), a novel class of relatively stable, highly lipophilic quinoline-based small-molecule inhibitors of DNA methyltransferase enzymes (DNMTs), into the culture medium of KFs. After a 24 h incubation period, we conducted a CCK-8 assay, which revealed a decrease in cell proliferation capability by 42.61 ± 8.54% (Fig. 3E). These findings suggest the involvement of DNA methylation in the abnormal growth of keloids, highlighting the importance of obtaining a comprehensive understanding of DNA methylation dynamics in keloid pathogenesis.

What’s more, literature has shown that DNMT3A plays a significant role in the functioning of SPOCD1 [18]. Thus, by employing plasmid transfection and si-RNA interference, we elevated and reduced the expression of DNMT3A. Subsequently, we observed that elevating DNMT3A decreased SPOCD1 expression in keloid fibroblasts. Conversely, reducing DNMT3A elevated SPOCD1 expression (Fig. 3F). This establishes a clear negative regulatory role between DNMT3A and SPOCD1, which is precisely how DNA methylation operates.

To further demonstrate the protein-protein interaction between DNMT3A and SPOCD1, we utilized co-immunoprecipitation (co-IP) technology. Co-IP is one of the simplest and most traditional method for studying protein-protein interactions. This assay is also one of the most reliable ones, since it does not require any chemical modification of either component in the complex (i.e., neither of the receptor nor of the arrestin). Therefore, it is the only assay that can detect and semi-quantify interactions between DNMT3A and other proteins. Co-IP of protein complexes from 10^7^ KFs cell lysates is used for mass spectrometry followed by western blotting using DNMT3A antibody (Fig. 2G). The entire system for mass spectrometry analysis is Q Active, which is connected in series with EASY-nanoLC 1000 ™ (Thermo Fisher Scientific, MA, USA). And the results are exhibit as. The mass spectrometry parameters are set as follows: (1) MS: scan range (m/z) =375–1500; Resolution=60,000; AGC target=3e6; maximum injection time=20 ms; include charge states=2-6; dynamic exclusion=30 s; (2) HCD-MS/MS: Resolution=15,000; isolation window=1.6; AGC target=1e5; maximum injection time=40 ms; collision energy = 25. Tandem mass spectrometry was analyzed by PEAKS Studio version X+ (Bioinformatics Solutions Inc., Waterloo, Canada) and present a reasonable protein ion peak graph (Fig. 3H). Furthermore, we found that SPOCD1 displays a prominent ion peak in the protein that DNMT3A has pulled down (Fig. 3I).

Combining the aforementioned results, we have substantial evidence to suggest a significant interaction between DNMT3A and SPOCD1.

### IFNγ could regulate the expression of DNMT3A in keloid

**Fig. 4:**
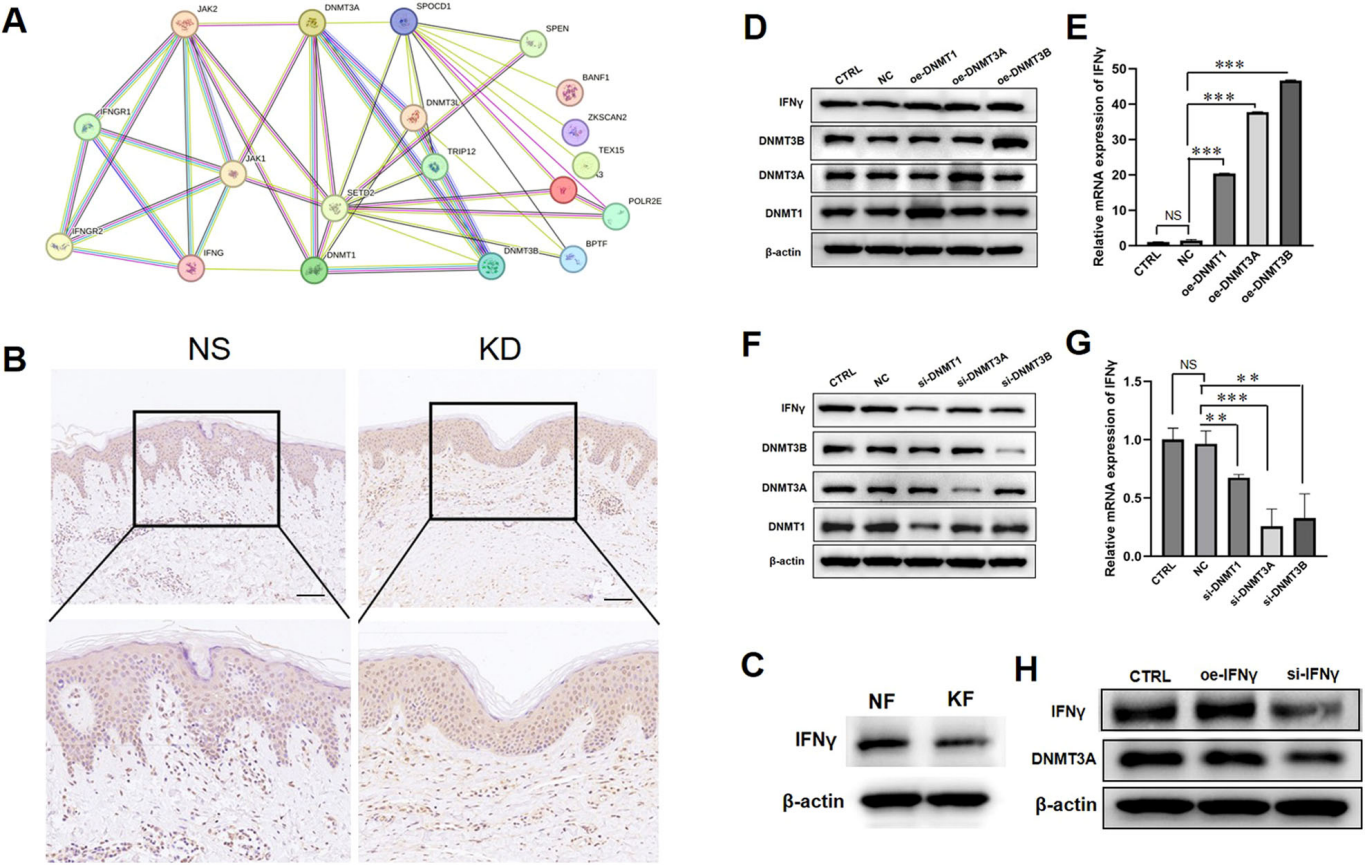
IFNγ could regulate the expression of DNMT3A in keloids. **A** The string of protein-protein interactions. **B** Immunohistochemical staining for IFNγ in normal skin and keloid. **C** The western blotting results of IFNγ in normal skin and keloid. **D** The western blotting results of KFs after treated with oe-DNMT1, oe-DNMT3A and oe-DNMT3B for 24 h. **E** Gene expression of KFs after treated with oe-DNMT1, oe-DNMT3A and oe-DNMT3B for 24 h. **F** The western blotting results of KFs after treated with si-DNMT1, si-DNMT3A and si-DNMT3B for 24 h. **G** Gene expression of KFs after treated with si-DNMT1, si-DNMT3A and si-DNMT3B for 24 h. **H** The western blotting results of IFNγ and DNMT3A after treated with si-IFNγ or oe-IFNγ. Bar=100 μm. *p < 0.05, **p < 0.01, ***p < 0.001.

In order to explore the relationship between including SPOCD1, DNMT3A and even other ferroptosis-related factors, we conducted protein-protein interaction analysis on the website of string and the protein-protein interaction map shows there is indeed an interactive relationship between SPOCD1 and DNMT3A and even IFNγ (Fig. 4A).

IFNγ is a homodimeric structure formed by the non-covalent bonding of two 17 kDa peptide subunits, primarily secreted by activated T cells, nearly all CD8 + T cells, and natural killer cells [19]. IFNγ operates through a cysteine/glutamic acid reverse transport system, solute carrier family 7 member 11(SLC7A11) and SLC3A2, that targets the surface of tumor cell membranes. This system reduces cysteine transport into cells during the body’s immune response, leading to decreased expression of glutathione (GSH) and glutathione peroxidase 4 (GPX4), thereby inducing ferroptosis in tumor cells and impeding tumor growth [20–22]. Studies have indicated that sorafenib, a ferroptosis inducer, could effectively inhibit KFs proliferation, ECM synthesis, and expression, while promoting KFs apoptosis and significantly increasing IFNγ expression in KFs [23]. What role does IFNγ play in keloid formation and progression?

This further motivates us to study the expression of IFNγ in keloid. As shown in Fig. 4B, the expression of IFNγ in KFs as well as in keloid is decreasing. We investigated the impact of IFNγ on KFs’ proliferation by treating KFs with varying concentrations of IFNγ and its monoclonal antibody (Emapalumab, Selleck, A204101). Our results, as shown in Suppl. Figs.5, revealed no significant differences in KFs proliferation rates. To delve deeper into the role of IFNγ in keloid development, we supplemented KFs supernatant with erastin, a ferroptosis inducer, along with IFNγ for a 24-hour period. it illustrates that additional IFNγ alone did not markedly decrease KFs proliferation rate, except when administered simultaneously with erastin (Suppl. Fig. 5C). As IFNγ has a regulatory function on ferroptosis, this suggests a potential role of IFNγ in regulating ferroptosis in KFs.

IFNγ has been reported to influence DNA methylation processes, potentially through regulating the expression or activity of DNMT3A. Specifically, IFNγ may modulate DNMT3A expression levels or activity, thereby impacting DNA methylation patterns across the genome [24, 25]. To reveal the relationship between IFNγ and DNMT3A, we hypothesized the DNMT family induced hypermethylation of IFNγ promoter in keloid so as to decrease its expression. As shown in Fig. 3, we found that over expression of DNMT3A, not DNMT1 or DNMT3B could not decrease the mRNA and protein levels of IFNγ (Fig. 4D, E). In comparison, knockdown DNMT3A significantly facilitated IFNγ expression (Fig. 4F, G). These results suggest a potential synergistic relationship between IFNγ and DNMT3A. Therefore, we observed the expression of DNMT3A through overexpression and interference of IFNγ expression. The results show that the expression of DNMT3A is synchronized with that of IFNγ (Fig. 4H).

In summary, we proposed our complete hypothesis that IFNγ may regulate SPOCD1 expression through DNMT3A.

### IFNγ could regulate the biological actives of KFs through SPOCD1

The above RNA-seq analysis has found that the expression of SPOCD1 which is involved in DNA methylation and its downstream genes was significantly increased in typical keloid samples. At the same time, DNMT3A can promote DNA methylation of SPOCD1 regulating, thereby reducing the expression of SPOCD1. What’s more, IFNγ has a synergistic relationship with DNMT3A in keloids. All these results suggest that IFNγ may regulate SPOCD1expression in keloid by DNA methylation indirectly, which in turn affects ferroptosis process regulated through IFNγ.

**Fig. 5:**
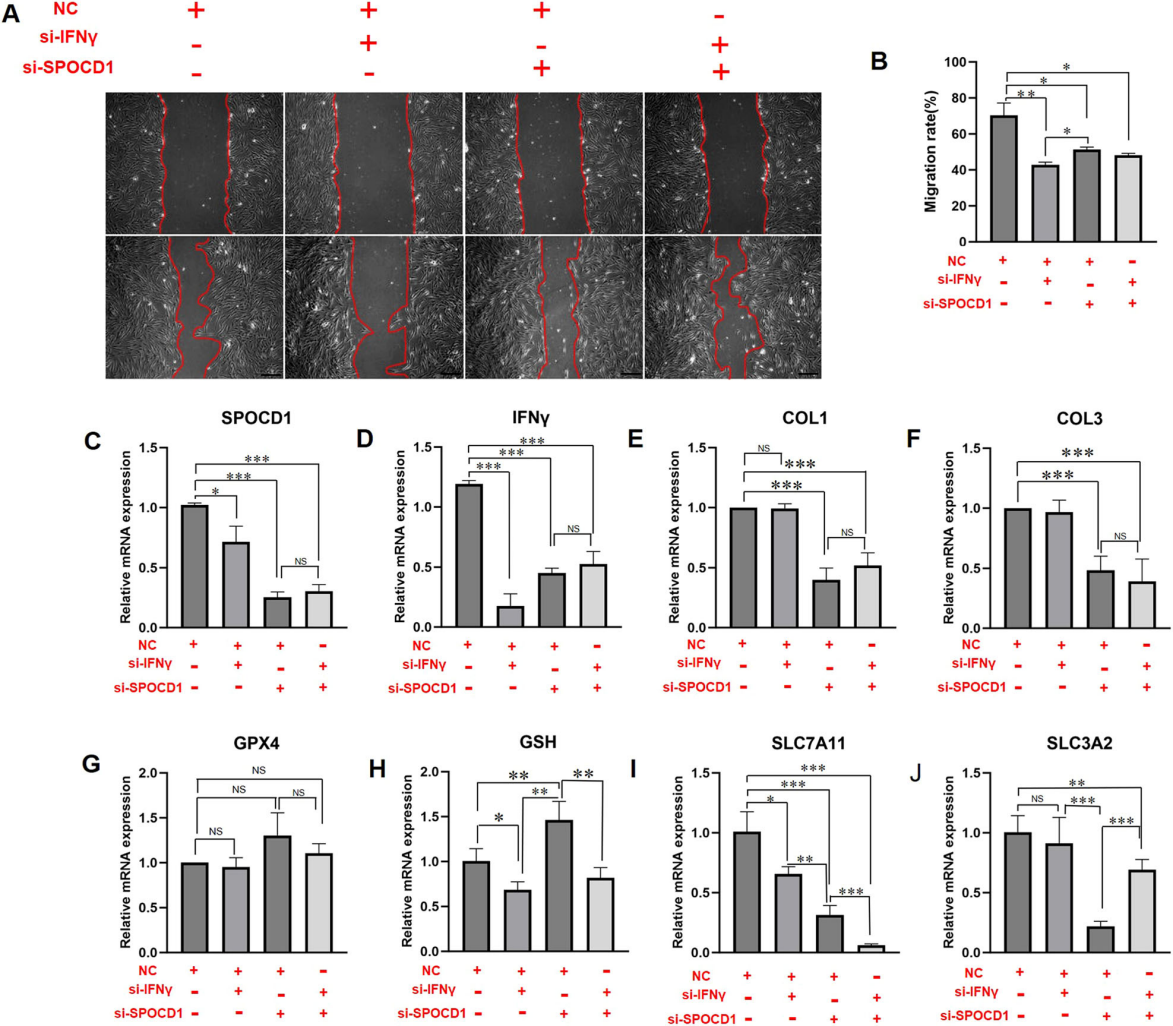
IFNγ could regulate the biological actives of KFs through SPOCD1. **A** The results of wound healing. **B** Quantitative analysis of wound healing model. **C**–**J** Gene expression of SPOCD1, IFNγ, col1, col3, GPX4, GSH, SLC7A11 and SLC3A2 in KFs treated with si-IFNγ, si-SPOCD1 or si-IFNγ + SPOCD1 for 48 h. Bar=100 μm. *p < 0.05, **p < 0.01, ***p < 0.001.

Subsequently, we designed three separate sets si-RNA of SPOCD1 and IFNγ and the siRNA sequence are shown in Supplemental material table 3. Two relatively effective sequence were selected for subsequent experiments. First, Wound healing experiments were performed to determine the effect of SPOCD1 and IFNγ on the migration of KFs. The results showed that all the experimental groups including si-IFNγ, si-SPOCD1 and si-IFNγ + SPOCD1, significantly inhibited the migration of KFs from 19.08 ± 5.172%to 27.49 ± 4.054% and si-SPOCD1 could deteriorate si-IFNγ’s inhibit effects partly (Fig. 5A, B).

From the results of RT-qPCR, si-IFNγ and si-SPOCD1 could effectively reduce the expression of IFNγ and SPOCD1 as both of them are decreased by over 70% (Fig. 5C, D). The decrease expression of IFNγ has no significant effect on the expression of col1 and col3 and these results align with previous research findings. Conversely, the decrease of SPOCD1 has significant inhibition on the expression of col1 and col3 which are the main extracellular matrix component of keloid (Fig. 5E, F). However, SPOCD1 participating in regulating ferroptosis of KFs was verified by 23% increases of GSH gene expression as SPOCD1 was interfered (Fig. 5H). The mRNA expression of SLC7A11 and SLC3A2 have a significant difference from ctrl, si-SPOCD1, si-IFNγ and si- IFNγ + SPOCD1 as si-SPOCD1 could rescue the promoting effects of si-IFNγ (Fig. 5H–J).

### IFNγ could regulate the ferroptosis of KFs through SPOCD1

**Fig. 6:**
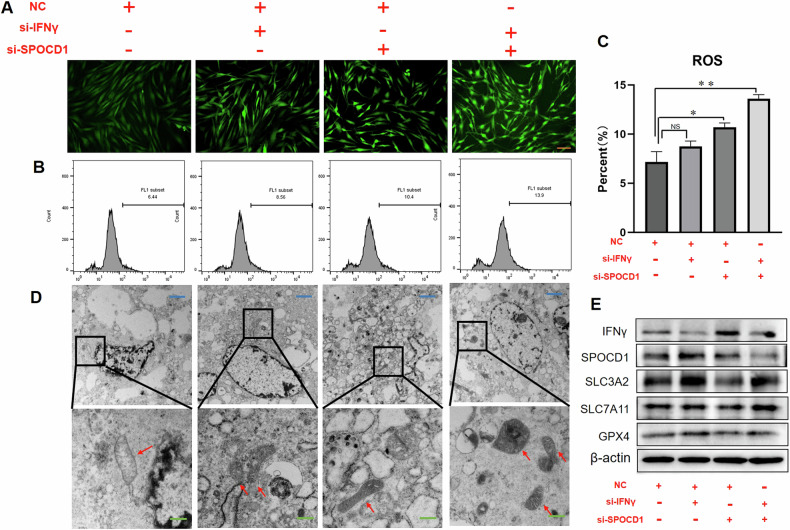
IFNγ could regulate the ferroptosis of KFs through SPOCD1. **A** Fluorescence intensity detection of ROS of KFs after treated with si-IFNγ, si-SPOCD1 or si-IFNγ + SPOCD1 for 48 h. **B** Flow analysis of ROS of KFs after treated with si-IFNγ, si-SPOCD1 or si-IFNγ + SPOCD1 for 48 h. **C** Quantitative statistics of ROS positive rate. **D**) Electron microscopy detection results of KFs treated with si-IFNγ, si-SPOCD1 or si-IFNγ + SPOCD1 for 72 h. **E** The western blotting results of KFs after treated with si-IFNγ, si-SPOCD1 or si-IFNγ + SPOCD1 for 72 h. Blue bar=2 μm, Green bar=500 nm, Orange bar=50 μm.*p < 0.05, **p < 0.01, ***p < 0.001.

During the occurrence of ferroptosis, cytokines often trigger an excessive production of ROS, leading to oxidative damage and mitochondrial dysfunctions [26]. Consequently, we conducted further investigations into the impact of different groups on intracellular ROS production. As shown in Fig. 6A, si-IFNγ has no significant effect on the green fluorescence inside the cells, indicating that ROS was not different from the NC groups. However, we observed a significant overproduction in the green signal within cells following si-SPOCD1 interventions (Fig. 6A-C). Compared to the control group, si-SPOCD1 + IFNγ also caused a significant increase in ROS, as the ROS-positive cell population increased 89.55 ± 7.94% (Fig. 6B). The same conclusion can be drawn from the results of MDA and GSSG/GSH(Suppl. Figs.6).

The most direct evidence of cell ferroptosis observed is the shrinkage of mitochondria and the rupture of cell membranes. Therefore, the treated cells were embedded and relevant electron microscopy results were obtained as shown in the Fig. 6D. The mitochondria in the control group were relatively plump, with abundant cytoplasm and intact mitochondrial membranes. The morphology of mitochondria in the si-IFNγ group was fair, with numerous mitochondria. Mitochondria in the si-SPOCD1 and si-IFNγ + SPOCD1 groups exhibited shrinkage, reduced cytoplasm, and some ruptured membranes. The evidence indicates that SPOCD1 can partially reverse IFNγ-induced ferroptosis.

Subsequently, we conducted Western blot analysis, as shown in Fig. 6E: the reduction of IFNγ effectively enhances the expression of SPOCD1 protein. Meanwhile, the decrease in SPOCD1 also reduces the expression of SLC7A11, SLC3A2 and GPX4, thereby reducing ferroptosis in KFs. Furthermore, si-IFNγ + SPOCD1 further promoted the protein expression of SLC7A11, SLC3A2 and GPX4, increasing cell ferroptosis, which is consistent with the aforementioned results.

### The prompting effect of SPOCD1 on keloid is approved by ex-vivo keloid explant culture

**Fig. 7:**
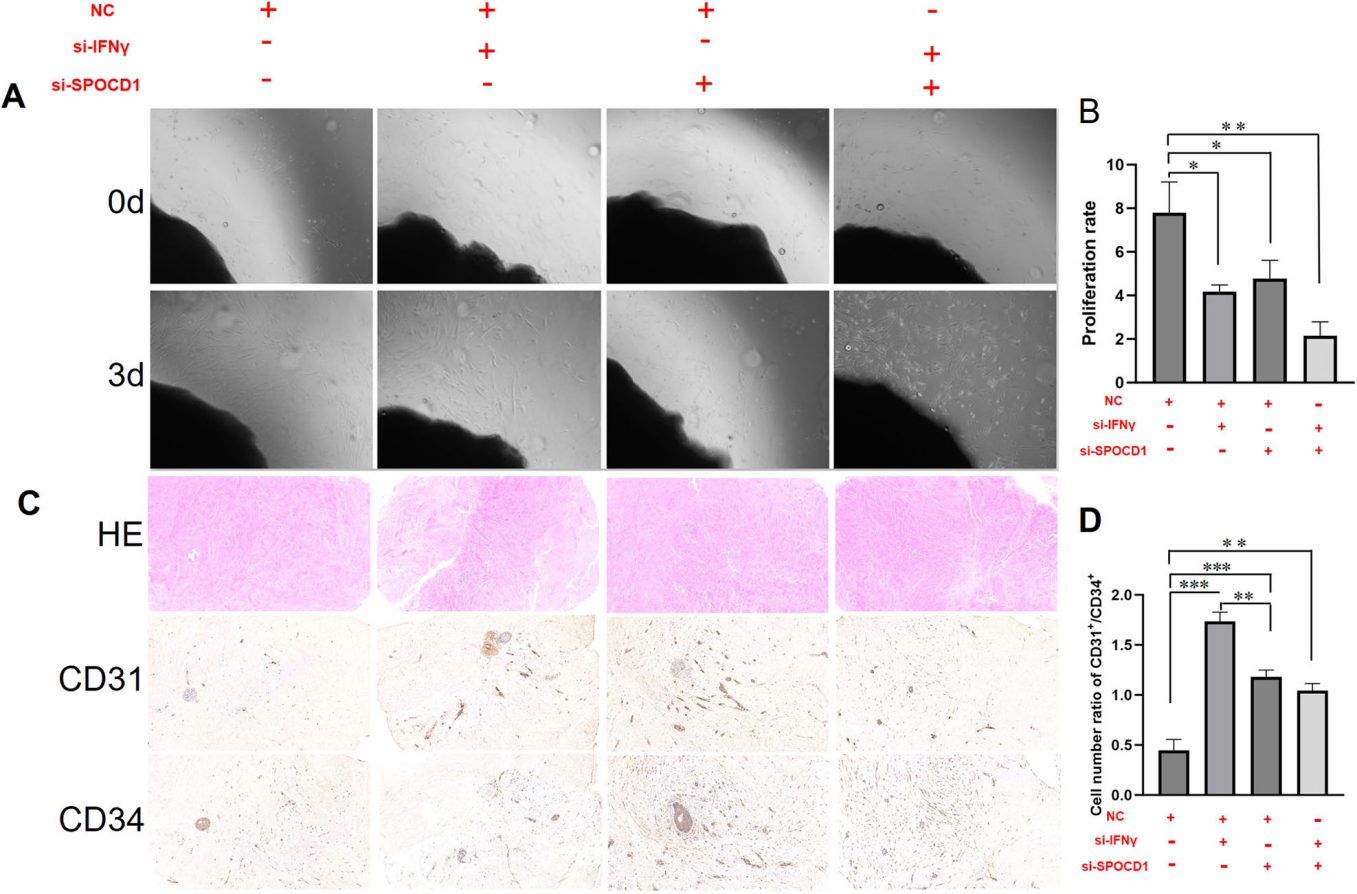
SPOCD1 could promote keloid’s growth approved by ex-vivo keloid explant culture. **A** Ex-vivo keloid explant culture at day 0 and day 3. **B** Quantitative analysis of cell proliferation in ex-vivo keloid explant culture. **C** *Hematoxylin-eosin staining* and immunohistochemical staining for CD31 and CD34 of ex-vivo keloid explant culture at day 3. **D** Quantitative analysis expression of CD31 and CD34.bar=100 μm. *p < 0.05, **p < 0.01, ***p < 0.001.

Ex-vivo keloid explant culture is frequently utilized as a model for keloid research in vitro [27]. Similarly, we utilized keloid tissue pieces for our in vitro study. We resected the keloid tissue pieces into 1-3 mm*1-3 mm pieces and planted them into a six-well plate. Once they had attached to the wall, we added DMEM containing 10% FBS for cultivation. The time when the cells uniformly spread out was considered day one, and we added si-IFNγ, si-SPOCD1 adn si-IFNγ + SPOCD1 for a further 3 days of cultivation. Then, the cells were photographed and counted. The experimental results are depicted in the Fig. 7A. Si-IFNγ and Si-SPOCD1 can effectively reduce the migration and proliferation rates of KFs, which is closely related to the biological regulatory effects of IFNγ and SPOCD1. Meanwhile, Si-IFNγ combined with si-SPOCD1 can further reduce the impact of si-IFNγ on keloid tissue, thereby reducing the cell migration rate (Fig. 7B).

Histologically, keloids contain an increased blood vessel density compared with normal dermis or normal scars [28, 29]. Hence, we wondered whether si-SPOCD1 would repress angiogenic activity. To better mimic the in vivo response to SPOCD1, human keloid explants were embedded and sectioned for further analyses. As demonstrated by HE staining (Fig. 7C), the collagen arrangement in the si-SPOCD1and si-IFNγ + SPOCD1 group is relatively more organized. As expected, we observed reduced cellularity and microvasculature in si-SPOCD1-treated tissue sections (Fig. 7C). In Masson and Sirius Red staining, it was observed that collagen deposition in the si IFN γ + SPOCD1 group decreased and became more disordered(Suppl. Figs.7). Quantification data revealed that approximately 2.87 ± 0.21 times of CD31^+^/ CD34^+^ cells were increased by si-IFNγ and 59% to 73% by si-SPOCD1 and si-IFNγ + SPOCD1 (Fig. 7D). CD31^+^ and CD34^+^ are both glycoproteins expressed on the surface of endothelial cells. CD34, a highly glycosylated type I transmembrane glycoprotein, is selectively expressed on the surface of human and other mammalian hematopoietic stem/progenitor cells and gradually decreases in expression with cellular maturation. Hence, the increase rate of CD31^+^/ CD34^+^ implies that si-SPOCD1 and si- IFNγ + SPOCD1 can diminish the stemness of vascular endothelial cells, curtail the neovascularization within the vascular endothelium of keloid, thereby inhibiting the growth of keloid.

## Discussion

Ferroptosis is a new type of programmed cell death that is iron dependent and distinct from apoptosis, necrosis, and autophagy [10]. The main mechanism of ferroptosis is that, under the action of divalent iron or ester oxygenase, it catalyzes the high expression of unsaturated fatty acids on the cell membrane, leading to lipid peroxidation and inducing cell death; Morphologically, it mainly manifests as a reduction (disappearance) of mitochondrial cristae, rupture and contraction of mitochondrial outer molds, and deep staining of mitochondrial colors; In molecular biology, the main manifestation is a decrease in the expression levels of ROS, SLC7A11/SLC3A2, GSH, and GPX4 [30, 31].

Few studies have focused on the ferroptosis of fibroblasts in keloid. Therefore, we aim to discover a cure for keloid through research on the ferroptosis of fibroblasts in keloid. We identified and confirmed that SPOCD1 is highly expressed in KD and KFs, an observation not previously documented in the literature. (Fig. 1D–F). We further discovered that SPOCD1 is closely linked with ferroptosis of KFs as it can inhibit KFs’ ferroptosis not through the canonical TGF-β1/smads pathway (Fig. 2).

According to literature reports [32, 33], DNA methylation is widely present in the regulation of gene expression. DNA methylation is one of the main regulatory mechanisms of epigenetics, which is related to immune regulation and inflammatory response in various diseases, such as systemic lupus erythematosus and rheumatoid arthritis [34]. DNA methylation leads to highly folded chromatin in corresponding regions of the genome, resulting in a decrease or even inactivation of gene transcription levels by inhibiting the binding of transcription factors and regulatory elements. Correspondingly, demethylation leads to a loose chromatin structure through facilitating the binding of transcription factors and regulatory elements and improving gene transcription levels [32]. Our research findings suggest that KD not only exhibits hypomethylation, but also shows a decrease in DNA methylation across various degrees for SPOCD1. This strongly indicates the significant role of DNA methylation and SPOCD1 in regulating KD’s growth (Fig. 3).

The ‘string’ database was utilized to predict potential regulators of DNMT3A and SPOCD1, with the results depicted in Fig. 4A. Notably, the role of IFNγ has captured our attention. The expression of IFNγ is reduced in keloid and KFs (Fig. 4B, C). IFNγ and ferroptosis are two distinct biological phenomena that have been studied extensively in the context of immune response and cell death, respectively. While they operate in different cellular pathways, there is evidence suggesting a relationship between IFNγ signaling and the regulation of ferroptosis. Some studies have suggested that IFNγ can induce ferroptosis in certain cell types [21]. For example, research has shown that IFNγ can enhance the sensitivity of cancer cells to ferroptosis inducers by upregulating the expression of iron transporters and promoting the accumulation of intracellular iron, thereby sensitizing cells to ferroptosis [20]. Additionally, the immune response mediated by IFNγ may indirectly influence ferroptosis in the context of inflammation and tissue damage. For instance, IFNγ-induced inflammation and oxidative stress can lead to the accumulation of lipid peroxides and contribute to ferroptosis in certain cell populations [35, 36]. Conversely, recent studies have also suggested that components of the ferroptosis pathway may regulate IFNγ signaling [37]. For example, the lipid peroxidation products generated during ferroptosis could modulate signaling pathways involved in immune responses, potentially impacting IFNγ-mediated signaling cascades [38, 39].

**Fig. 8:**
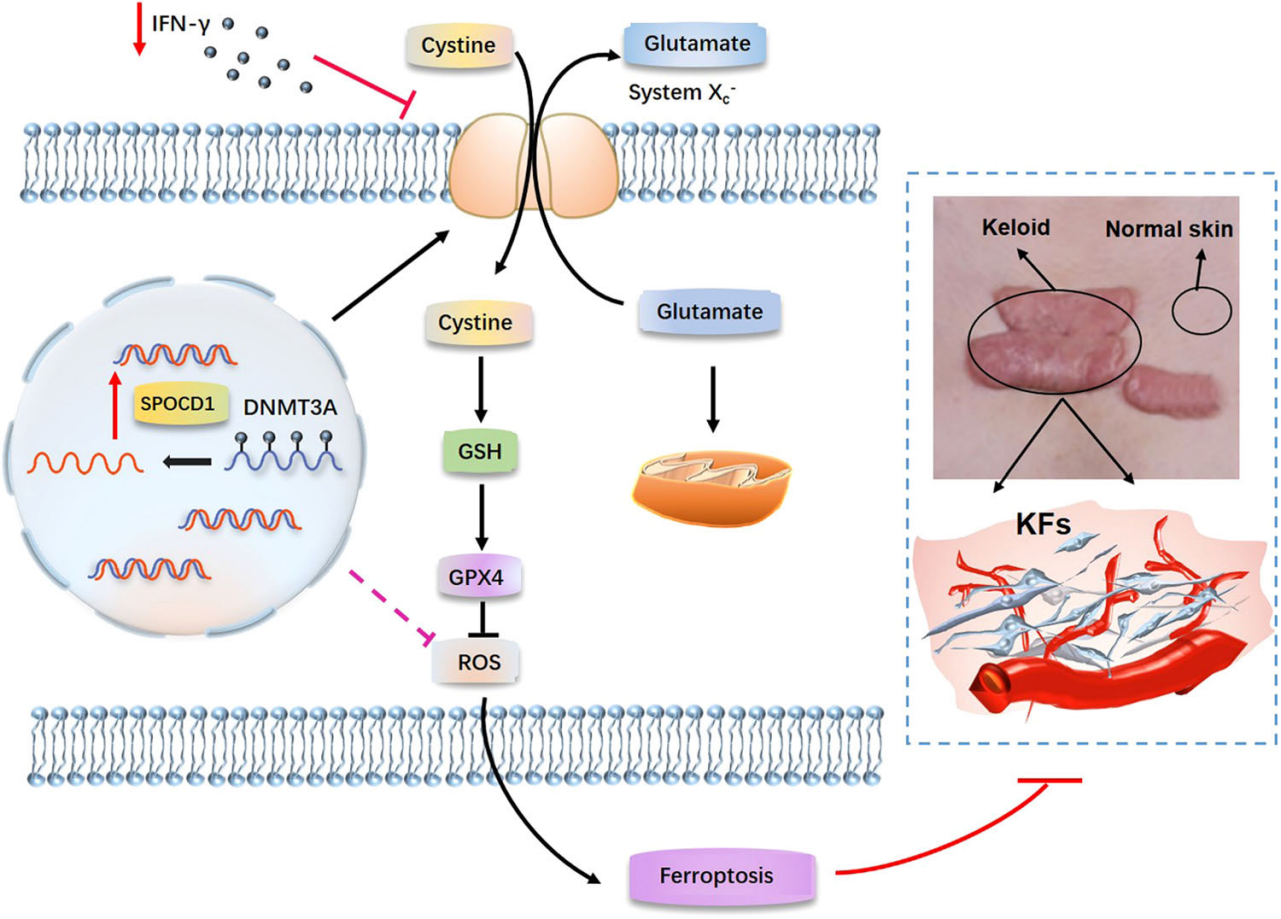
IFNγ regulates ferroptosis in KFs by inhibiting the expression of SPOCD1 through DNMT3A. Knockdown IFNγ induces a decrease in DNMT3A, which in turn increase SPOCD1 expression in KFs, then leads to col1, col3 excess and ROS reduction, and ultimately decreases KFs ferroptosis and ECM deposition. *p < 0.05, **p < 0.01, ***p < 0.001.

Several literatures reports, the relationship between IFNγ and DNA methylation is bidirectional and context-dependent, with IFNγ influencing DNA methylation patterns and DNA methylation regulating IFNγ signaling and immune responses [40, 41]. And during the proliferation, apoptosis, migration, and differentiation of keloid, multiple genes is involved in methylation, which may be related to hormonal changes and immune regulation in the body [42, 43]. Hence, we hypothesized that the expression of IFNγ in keloid was also related with DNA methylation. However, the expression of IFNγ is consistent with that of DNMT3A, implying that an elevation in DNMT3A expression can also induce an elevation in IFNγ, and vice versa (Fig. 4D–H). This suggests a coordinated role between the two in keloid. In light of the regulatory role of DNMT3A on SPOCD1, we speculate that IFNγ can modulate the expression of SPOCD1 via DNMT3A, and in turn, regulate ferroptosis in KFs (Fig. 8).

Subsequently, we designed a series of rescue experiments to test our hypothesis. As shown in Fig. 5, si-SPOCD1 can partially inhibit the migration of KFs as well as the expression of col1 and col3, and si-IFNγ + SPOCD1 also partially reverses the effect of si-IFNγ. Similarly, the expression of GSH and SLC7A11, the detection of ROS, and the results of electron microscopy also show similar patterns (Fig. 5H, J, Fig. 6). These results demonstrate that SPOCD1 may regulate ferroptosis by promoting the expression of SLC7A11 and inhibiting the generation of ROS. Owing to the scarcity of effective animal models in the study of keloid formation, we frequently employ in vitro keloid tissue culture methods to replicate in vivo experiments. In these simulations, we have also confirmed the inhibitory impact of si-SPOCD1 on keloid growth, diminishing the propensity for KD to form new blood vessels, and the modulation of IFNγ on SPOCD1(Fig. 7). The negative results of Ki-67 staining (Suppl. Figs.7) indicated that keloid differs from tumors and possesses their own unique growth patterns and mechanisms. The regulatory relationship between IFNγ and SPOCD1 may emerge as a novel target for treating keloid and preventing their recurrence.

CD8^+^T cells are the major source of IFNγ production [44, 45]. Previous studies have indicated that the IFNγ produced by CD8^+^T cells appears to improve antitumor responses by enhancing tumor sensitivity to ferroptosis [46, 47]. And the development of keloid is also influenced by the modulation of immune responses [48–50]. Chen et al. used flow cytometry to analyze the subsets and functions of cells in keloid and found that the number of effector memory CD8^+^T cells and CD103^+^CD8^+^ resident memory T cells was increased in keloid tissue [51]. Studies investigating the role of CD8^+^T cells in the pathogenesis of keloids are lacking. Our research serves as an important piece of evidence for CD8 + T cells in modulating the keloid immune response, and it also represents a critical approach for our future keloid treatment.

## Materials And Methods

### Ethics statement

All protocols of the current study were performed with approval of the ethics committee of Shanghai ninth people’s hospital and all patients had provided informed content.

### Human subjects

A total of 35 keloid samples (Suppl. table S1) were collected from the department of plastic and reconstructive surgery of Shanghai ninth people’s hospital during March 2021 to January 2023. The patients (aged 22–52 years with a mean age of 30.57 ± 7.55 years) were pathologically diagnosed as Keloid. The normal skin tissues were set as negative control.

### Cell Treatment

Keloid fibroblasts from 35 keloid samples were obtained after excision from the corresponding sites. The process of cell extraction and cultivation is as described in the literature [7, 8]. The cells with the lower expression of SPOCD1 or IFNγ was screened by qRT-PCR. First, the KFs was seeded into a six-well cell culture plate (4 × 105 cells/well). After 24 h of culture, they were further cultured in DNMT inhibitor 5-aza-dc medium (the final concentration was 2 mmol/L), and the culture medium was changed every other day. Second, the KFs was seeded in a six-well cell culture plate at a density of 4 × 105 cells/well. Si-RNA containing SPOCD1, IFNγ, DNMT1, DNMT3A and DNMT3B (GenePharma, Shanghai, China) were diluted into 200 pmol using 250 mL serum-free Opti-MEM. Subsequently, 5 μL Lipofectamine™ 2000 (ThermoFisher Scientific, America) were added into 250 μL DMEM. Then, the solution of si-RNA and Lipofectamine™ 2000 were mixed equivoluminously and incubated at room temperature for 15 minutes. After adding 1.5 mL DMEM and 500 μL the above mixture, relative testing was conducted following 72 h of cultivation.

### qRT-PCR

The cells were collected and lysed using EZ-press RNA Purification Kit (B0004D) (EZBioscience, USA) to extract the total RNA. A Color SYBR Green qPCR Mix (A0012-R2) (EZBioscience, USA) was adopted to reverse transcribe the extracted RNA. The target gene was quantitatively analyzed by fluorescence qPCR with reference to SYBR Premix. Real-time fluorescence qPCR was performed using a Thermal Cycler Dice Real Time System amplifier (TP800; Takara Holdings, Kyoto, Japan). Primer sequences (Suppl. table S2) were synthesized by Sangon Biotech (Shanghai, China), with glyceraldehyde-3-phosphate dehydrogenase (GAPDH) used as internal reference gene. The fold changes were calculated using the 2-DDCt method.

### Western Blot Analysis

The total cell protein was isolated using the radioimmunoprecipitation assay IV (RIPA IV) lysate (R0010; Beijing Solarbio Science & Technology, Beijing, China). The protein was transferred onto a 0.45μm polyvinylidene fluoride (PVDF) membrane and then immunoblotted. The primary antibodies included rabbit antibodies against β-actin (proteintech, 1:1,000), SPOCD1 (proteintech, 1:5,000), IFNγ (proteintech, 1:1,000), GPX4 (proteintech, 1:10,000), GSH (proteintech, 1:1,000), SLC7A11 (proteintech, 1:1,000), SLC3A2 (proteintech, 1:1,000), DNMT1 (proteintech, 1:1,000), DNMT3A (proteintech, 1:1,000), and DNMT3B (proteintech, 1:2,000). The secondary antibody included horseradish peroxidase (HRP)-labeled goat anti-rabbit immunoglobulin G (IgG; proteintech, 1:10000).

### Transmission electron microscopy

Treated cells cultured in 6-cm dishes were fixed with a solution containing 2.5% glutaraldehyde. After being washed in 0.1 M phosphate buffer (pH 7.4) three times, cells were postfixed with phosphate buffer containing 1% osmic acid, followed by washing in 0.1 M phosphate buffer (pH 7.4) another three times. After dehydration and embedding, samples were incubated in a 60 °C oven for 48 h. Ultrathin sections were prepared and stained with lead citrate and uranyl acetate. After drying overnight, the sections were examined with a Hitachi transmission electron microscope (Hitachi, Japan).

### Cell viability assays

For cell proliferation assays, 2000 cells per well were seeded in quintuplicate in 96-well plates and incubated for 24 h. The cells were seeded at a density of 1000 cells per well and incubated for 0, 24, 48, 72 and 96 at 37 °C. Cell viability was measured with a CCK-kit (Beyotime) according to the manufacturer’s instructions. Absorbance was measured at an optical density of 562 nm in a spectrophotometric plate reader with a reference wavelength of 450 nm.

### Co-Immunoprecipitation (Co-IP)

For Co-IP assays, DSP (Thermo Fisher, Cat. 22585) was used to cross-link interacting proteins. After the cross-link, whole cell lysate was obtained using a RIPA buffer supplemented with PMSF, the protease inhibitor cocktail (Sigma–Aldrich, Cat. S8830), and phosphosite (Roche, Cat. 4906845001, Switzerland) followed by sonication and centrifugation at 12,000 rpm at 4 °C for 10 min. IgG and A/G agarose (Thermo Fisher, Cat. 20421) were used to pre-clean the cell lysate before IP with antibodies against DNMT3A overnight at 4 °C with gentle agitation. Then, 40 μl prewashed protein was added and incubated at a temperature of 4 h with gentle agitation. After extensive washing with an IP buffer twice and with PBS, the interacting proteins were eluted with SDS-PAGE.

### RNA-Seq and bioinformatics analysis

TRIzol reagent was utilized for total KD or normal skin tissue RNA extraction, and RNA purity assessment, quantification, and transcriptome library construction were carried out. OE Biotech Co., LTD (Shanghai, China) conducted the analysis and sequencing of the transcriptome. The Illumina Novaseq 6000 platform was used to sequence the libraries, producing 150 bp paired-end reads. DESeq2 software was applied for differential gene expression analysis with a consistent q-value threshold of ≤ 0.05 and fold change criteria of > 2 or groups and samples. Subsequently, Using the hypergeometric distribution algorithm, GO and KEGG Pathway enrichment analyses were conducted on the DEGs to identify significantly enriched functional terms. The GSEA software was utilized to conduct gene set enrichment analysis. The corresponding results have been uploaded to the GEO repository, with the serial number GSE280420.

### ROS generation detection

For ROS detection, NFs or KFs cells were treated with IFNγ or si-RNA as described above. After another 12 h incubation, cells were treated with laser irradiation and then stained with 2,7-dichlorodihydrofluorescein diacetate (DCFH-DA, in green) and incubated at 37 °C for 1 h, followed by CLSM and flow cytometry analysis.

### DNA methylation assay

Genomic DNA was extracted from human keloid or normal skin employing the QIAampTM DNA and Blood Mini kit (Qiagen) referring to the manufacturer’s protocol. All the testing procedures are carried out by OE Biotech Co., Ltd (Shanghai, China). We used the normalized data to compute the DNA methylation levels, displaying β values ranging from 0 to 1, corresponding to unmethylated and methylated sites, separately. We performed cluster analysis of differentially methylated sites and analyzed biological function. The corresponding results have been uploaded to the GEO repository, with the serial numbers SAMN44486717, SAMN44486718, SAMN44486719, SAMN44486720, SAMN44486721 and SAMN44486722.

### Immunofluorescence

The cell lines were plated onto cover-slips and treated, respectively. After 48 h, cells were fixed with 4% paraformaldehyde for 10 min at room temperature. All slides were then washed with PBS and blocked with Goat serum at room temperature for 30 min. Cells were incubated with primary antibodies (SPOCD1: proteintech) at 4 °C overnight, washed with PBS, and then incubated with appropriated secondary antibodies for 1 h at room temperature. Cells were examined immediately using Nikon C-HGFI Intensilight Fiber Illuminator (Nikon, Japan) fluorescence microscope.

### Histological, immunohistochemical, masson and sirius red analysis

The keloid specimens were fixed in 4% paraformaldehyde at 4 °C overnight, embedded in paraffin blocks, and sectioned to 5 μm thicknesses. The sections were stained with hematoxylin and eosin (H&E) for routine examination. In addition, the keloid sections were incubated with antibodies against IFNγ, CD31 and CD34 at a dilution of 1:500. The bound antibodies were eventually visualized using 3,3′-diaminobenzidine (DAB) as a chromogen (Dako), and the slides were counterstained with hematoxylin. The numbers of CD31+ and CD34+ vessels were obtained in six randomly selected fields under the microscope. At the same time, the specimens were also subjected to Masson trichrome staining and Sirius red staining to observe collagen deposition.

### Malondialdehyde (MDA) assay

Treat transfected cells according to the instruction manuals for the Malondialdehyde (MDA) Detection Kit (Beyotime, China) and the level of MDA was presented as μmol/g protein.

### Glutathione (GSH) /GSSG assay

Briefly, cell samples were processed according to GSH Assay Kit (Beyotime, China) operating manual, absorbance was measured with an enzyme marker A412. The content of GSH was calculated from the measured total glutathione content and GSSG content according to the following formula: GSH=Total Glutathione-GSSG×2. The results are presented in the ratio of GSSH/GSH.

### Statistical analysis

The GraphPad Prism 10.0 was used for statistical analysis. The results were presented as mean ± SD or mean ± SEM. Student t-test, One-Way ANOVA, or Two-Way ANOVA were employed for statistical comparison. Statistical significance was determined with 95% (*p < 0.05), 99% (**p < 0.01) and 99.9% (***p < 0.001) confidence intervals.

## Data Availability

RNA-sequencing data are available through Gene Expression Omnibus accession number GSE280420. The rest are reflected in the Materials and Methods section.

## Suppl. information

Suppl. table and figure legends
Suppl. table s1
Suppl. table s2
Suppl. table s3
Suppl. Fig. s4
Suppl. Fig. s5
Suppl. Fig. s6
Suppl. Fig. s7
Suppl. western blot
