# Supplementary material for: IFNγ regulates ferroptosis in KFs by inhibiting the expression of SPOCD1 through DNMT3A
Source: Cell Death Discov. 2025 Jan 16;11:9. doi: 10.1038/s41420-024-02257-z (PMC11739694; doi:10.1038/s41420-024-02257-z)
Supplement: Supplementary file 1 — Suppl. table and figure legends [file 41420_2024_2257_MOESM1_ESM.docx]

**Supplementary Table S1**: Demographic data of keloid samples used in this study.

**Supplementary Table S2:** Primers used in quantitative PCR analysis.

**Supplementary Table S3:** The sequences of si-RNA. DNMT1-S3, DNMT3A-S3, DNMT3B-S3, SPOCD1-S3 and IFN-r-S4 were the actual sequence in use.

**Supplementary figure S4:** The levels of total glutathione and GSSG in the KFs treated with si-SPOCD1 or Erastin were measured sequentially using the GSH/GSSG assay kit with an enzyme marker, and then the levels of GSH were calculated according to the formula.

**Supplementary figure S5:** IFN-γ has no effect on the proliferation of KFs. A, B) The impact of varying concentrations of IFN-γ and its antibodies on the proliferation of KFs. C) The study investigates the impact of IFN-γ(10ng/ml), an ferr-inducer(erastin, 2.5μM), and an ferr-inhibitor(ferrostatin, 1μM) on the proliferation of KFs using CCK8.

**Supplementary figure S6:** A. The amount of MDA in the KFs treated with si-SPOCD1, si-IFNγ or both was detected using the MDA assay kit and measured with an enzyme marker. B. The levels of total glutathione and GSSG in the KFs treated with si-SPOCD1, si-IFNγ or both were measured sequentially using the GSH/GSSG assay kit with an enzyme marker, and the results were shown as GSSG/GSH.

**Supplementary figure S7:** masson, ki-67and sirius red staining of ex-vivo keloid explant.
