# Supplementary material for: IFNγ regulates ferroptosis in KFs by inhibiting the expression of SPOCD1 through DNMT3A
Source: Cell Death Discov. 2025 Jan 16;11:9. doi: 10.1038/s41420-024-02257-z (PMC11739694; doi:10.1038/s41420-024-02257-z)
Supplement: Supplementary file 2 — Suppl. table s1 [file 41420_2024_2257_MOESM2_ESM.docx]

**Supplementary Table S1**: Demographic data of keloid samples used in this study.

Sample No. Gender Ethnic Background Age Site of Specimen

KD1 M Asian 32 Chest

KD2 M Asian 43 Chest

KD3 M Asian 29 Chest

KD4 M Asian 25 Chest

KD5 F Asian 33 Chest

KD6 M Asian 32 Chest

KD7 M Asian 27 Chest

KD8 F Asian 33 Chest

KD9 M Asian 35 Chest

KD10 F Asian 29 Chest

KD11 F Asian 25 Chest

KD12 F Asian 27 Chest

KD13 M Asian 38 Chest

KD14 M Asian 22 Chest

KD15 F Asian 26 Chest

KD16 F Asian 29 Chest

KD17 F Asian 25 Chest

KD18 M Asian 27 Chest

KD19 F Asian 42 Chest

KD20 F Asian 33 Chest

KD21 F Asian 52 Chest

KD22 F Asian 32 Chest

KD23 M Asian 29 Chest

KD24 F Asian 22 Chest

KD25 F Asian 28 Chest

KD26 F Asian 23 Chest

KD27 F Asian 33 Chest

KD28 M Asian 22 Chest

KD29 F Asian 24 Chest

KD30 M Asian 40 Chest

KD31 F Asian 23 Chest

KD32 F Asian 31 Chest

KD33 M Asian 26 Chest

KD34 F Asian 23 Chest

KD35 M Asian 50 Chest

Note: Samples KD31-KD35 were used for ex-vivo keloid explant culture.
