# Supplementary material for: IFNγ regulates ferroptosis in KFs by inhibiting the expression of SPOCD1 through DNMT3A
Source: Cell Death Discov. 2025 Jan 16;11:9. doi: 10.1038/s41420-024-02257-z (PMC11739694; doi:10.1038/s41420-024-02257-z)
Supplement: Supplementary file 3 — Suppl. table s2 [file 41420_2024_2257_MOESM3_ESM.docx]

**Supplementary Table S2:** Primers used in quantitative PCR analysis.

Gene Name Primer Sequence (5’-3’)

Collagen I Sense: GGCGGCCAGGGCTCCGACCC

Antisense:AATTCCTGGTCTGGGGCACC

Collagen III Sense: TGGTGTTGGAGCCGCTGCCA

Antisense:CTCAGCACTAGAATCTGTCC

TGF-β1 Sense: AAGGACCTCGGCTGGAAGTG

Antisense: CCGGGTTATGCTGGTTGTA

SPOCD1 Sense: CTCCCCAAGTTGCTGACCTG

Antisense: CCCCTGTAGCGGGCAAATATC

IFNγ Sense: TGGGTTGTGTGTTTATTTCACT

Antisense: CATTAAAGCACTGGCTCAGAT

DNMT3A Sense: GCAGGATAGCCAAGTTCAG

Antisense: AATACCCTTTCCATTTCAGTG

DNMT3B Sense: CGAATTTTACCACCTGCTGAATT

Antisense: AGAACGGCCGGTCATCAC

DNMT1 Sense: CCTCCAAAAACCCAGCCAAC

Antisense: TCCAGGACCCTGGGGATTTC

SLC7a11 Sense: TGCTGGGCTGATTTTATCTTCG

Antisense: GAAAGGGCAACCATGAAGAGG

SLC3a2 Sense: CTGGTGCCGTGGTCATAATC

Antisense: GCTCAGGTAATCGAGACGCC

GPX4 Sense: CGATACGCCGAGTGTGGTTTAC

Antisense: ACAGCCGTTCTTGTCAATGAGG

GSH Sense: GACCAGCGTGCCATAGAGA

Antisense: CCAGAGACCCCTTTTCAGAGAT

GAPDH Sense: TCACCATCTTCCAGGAGCG

Antisense: CTGCTTCACCACCTTCTTGA
