# Supplementary material for: IFNγ regulates ferroptosis in KFs by inhibiting the expression of SPOCD1 through DNMT3A
Source: Cell Death Discov. 2025 Jan 16;11:9. doi: 10.1038/s41420-024-02257-z (PMC11739694; doi:10.1038/s41420-024-02257-z)
Supplement: Supplementary file 4 — Suppl. table s3 [file 41420_2024_2257_MOESM4_ESM.docx]

**Supplementary Table S3:** The sequences of si-RNA. DNMT1-S3, DNMT3A-S3, DNMT3B-S3, SPOCD1-S3 and IFN-r-S4 were the actual sequence in use.

|  |  |  |  |
| --- | --- | --- | --- |
| **gene name** | **numbers** | **sequences** | |
|  |  | **sense（5'-3'）** | **antisense（5'-3'）** |
| **human DNMT1** | DNMT1-S1 | GGGACUGUGUCUCUGUUAUTT | AUAACAGAGACACAGUCCCTT |
|  | DNMT1-S2 | GAGGACAACAAGUUCAAAUTT | AUUUGAACUUGUUGUCCUCTT |
|  | DNMT1-S3 | GAGGCCUAUAAUGCAAAGATT | UCUUUGCAUUAUAGGCCUCTT |
| **human DNMT3A** | DNMT3A-S1 | GCGUCACACAGAAGCAUAUTT | AUAUGCUUCUGUGUGACGCTT |
|  | DNMT3A-S2 | GGCUCUUCUUUGAGUUCUATT | UAGAACUCAAAGAAGAGCCTT |
|  | DNMT3A-S3 | GUCCACUAUACUGACGUCUTT | AGACGUCAGUAUAGUGGACTT |
| **human DNMT3B** | DNMT3B-S1 | CACGCAACCAGUGGUUAAUTT | AUUAACCACUGGUUGCGUGTT |
|  | DNMT3B-S2 | CGCCUCAAGACAAAUUGCUTT | AGCAAUUUGUCUUGAGGCGTT |
|  | DNMT3B-S3 | GCCCAUUUGACUUGGUGAUTT | AUCACCAAGUCAAAUGGGCTT |
| **human SPOCD1** | SPOCD1-S1 | CAGAGACGGUGGAAUGAGUUCUCCA | UGGAGAACUCAUUCCACCGUCUCUG |
|  | SPOCD1-S2 | CCAAGUAUCGCAGCCUGCUGUUCAA | UUGAACAGCAGGCUGCGAUACUUGG |
|  | SPOCD1-S3 | CAGUAAGGUGGAGAAGAGAUACUAU | AUAGUAUCUCUUCUCCACCUUACUG |
| **human IFN-r** | IFN-r-S4 | UGGAGACCAUCAAGGAAGACAUGAA | UUCAUGUCUUCCUUGAUGGUCUCCA |
