# Supplementary figures and images for: IFNγ regulates ferroptosis in KFs by inhibiting the expression of SPOCD1 through DNMT3A
Source: Cell Death Discov. 2025 Jan 16;11:9. doi: 10.1038/s41420-024-02257-z (PMC11739694; doi:10.1038/s41420-024-02257-z)

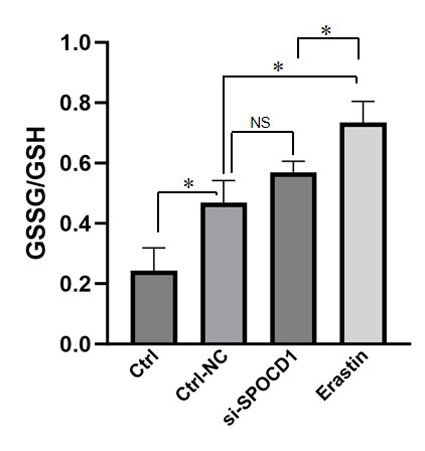

Supplement: Supplementary file 5 — Suppl. Fig. s4 [file 41420_2024_2257_MOESM5_ESM.jpg]

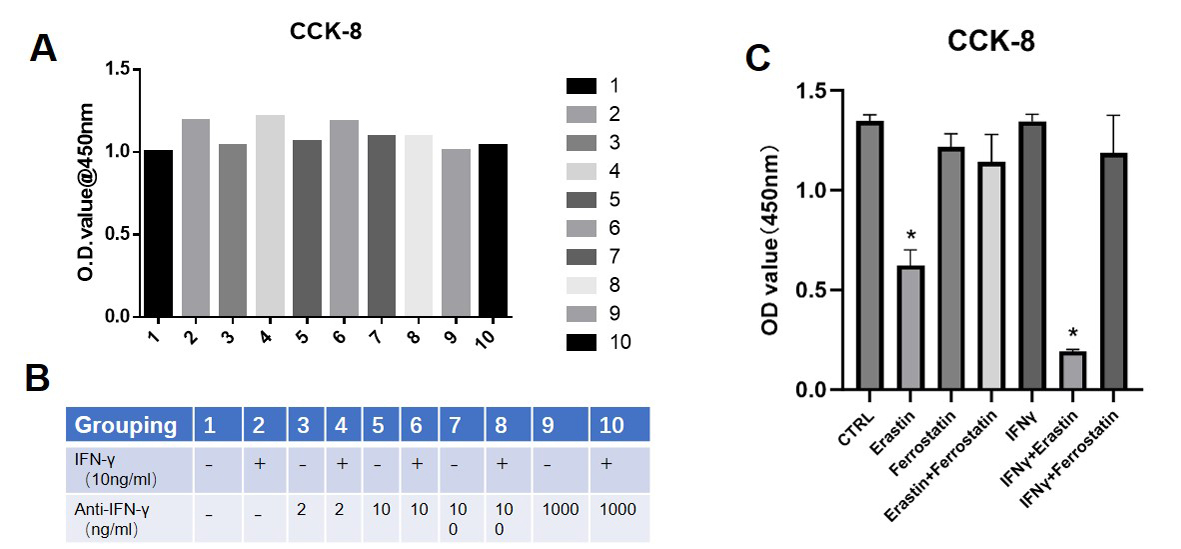

Supplement: Supplementary file 6 — Suppl. Fig. s5 [file 41420_2024_2257_MOESM6_ESM.jpg]

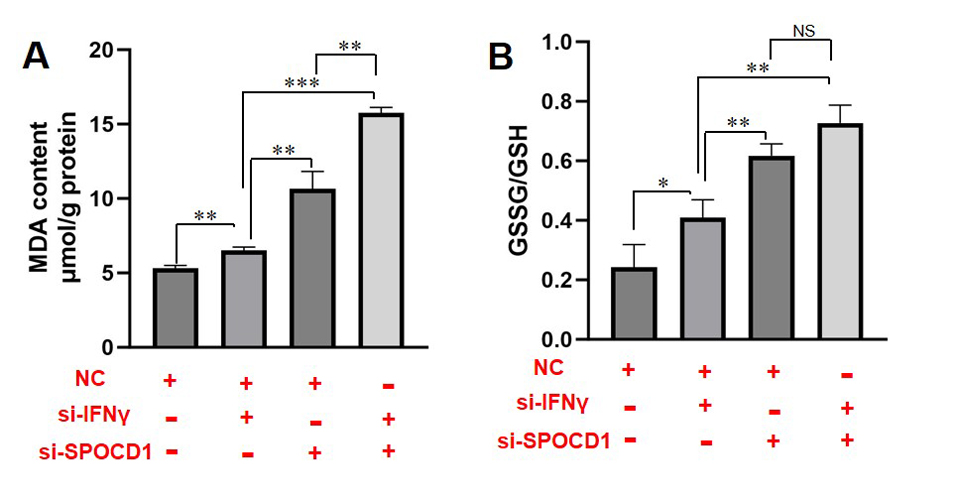

Supplement: Supplementary file 7 — Suppl. Fig. s6 [file 41420_2024_2257_MOESM7_ESM.jpg]

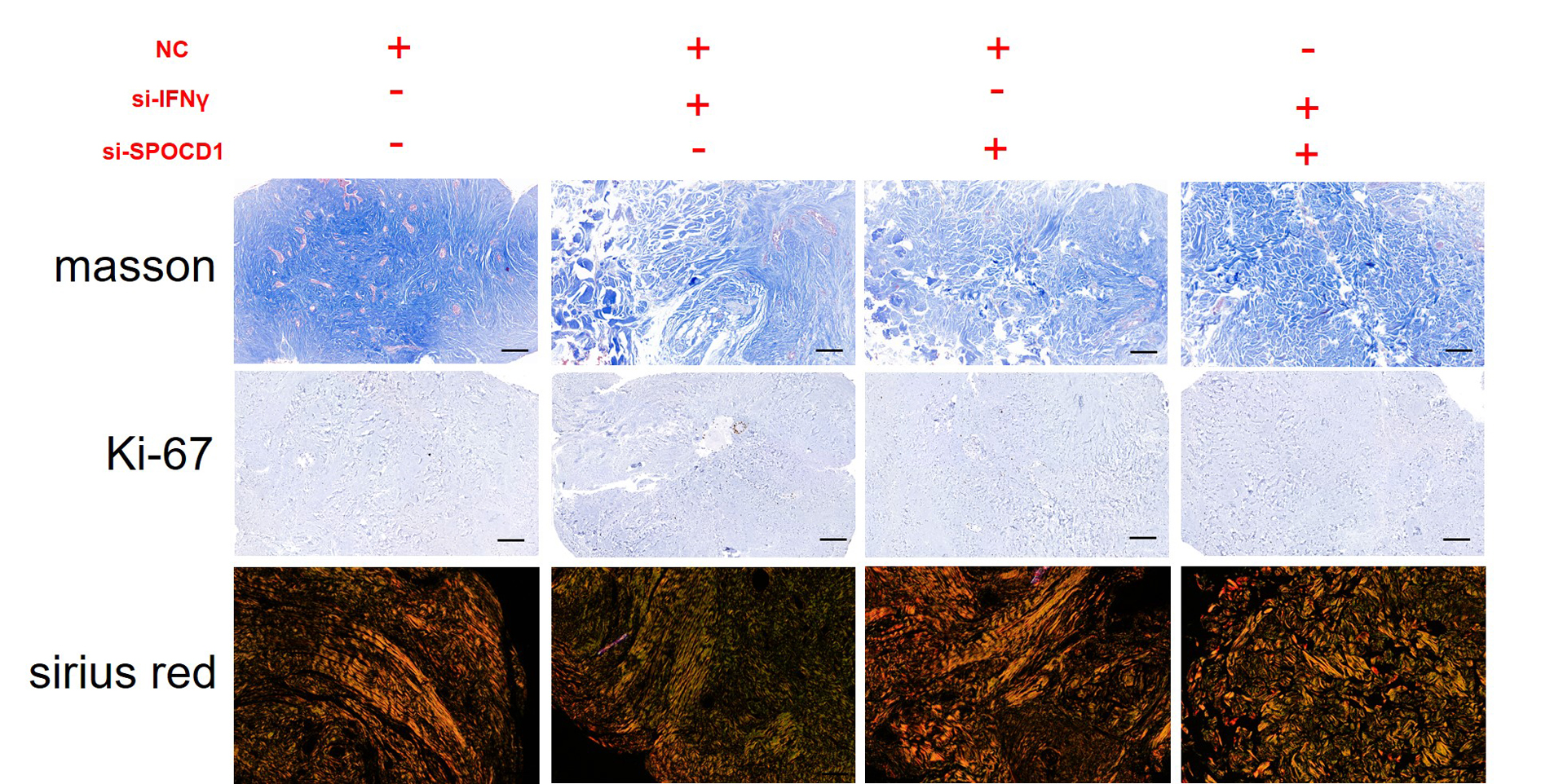

Supplement: Supplementary file 8 — Suppl. Fig. s7 [file 41420_2024_2257_MOESM8_ESM.jpg]

Figure 1

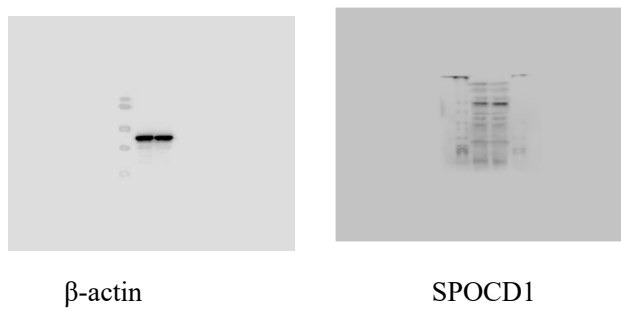

Figure 3F

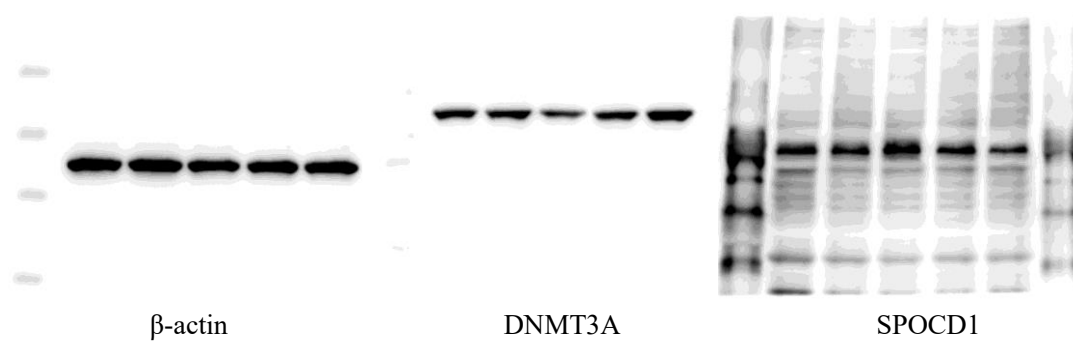

Figure 3G

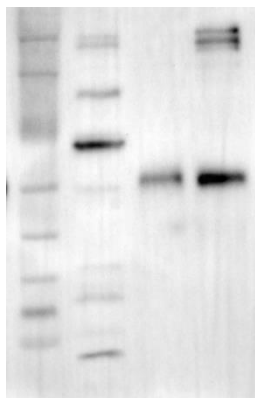

Figure 4D

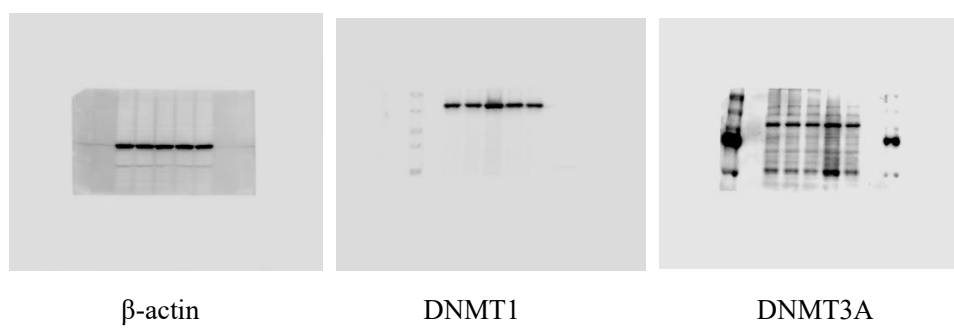

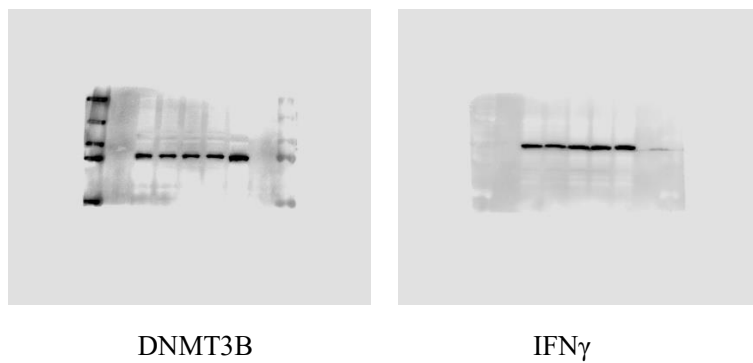

Figure 4F

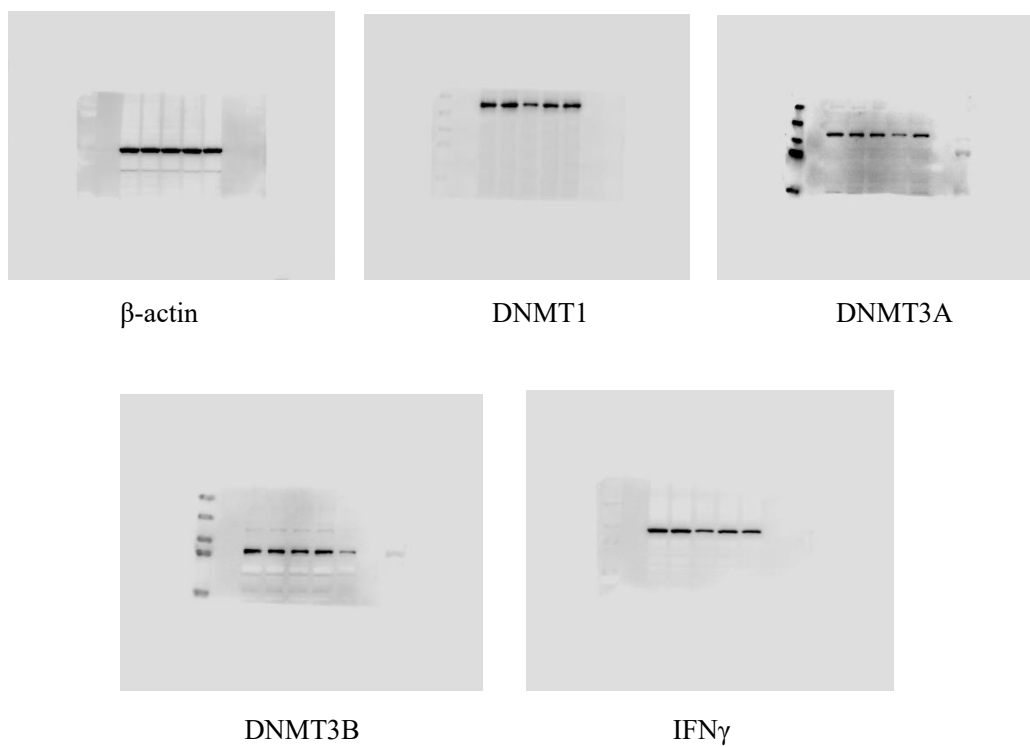

Figure 4C

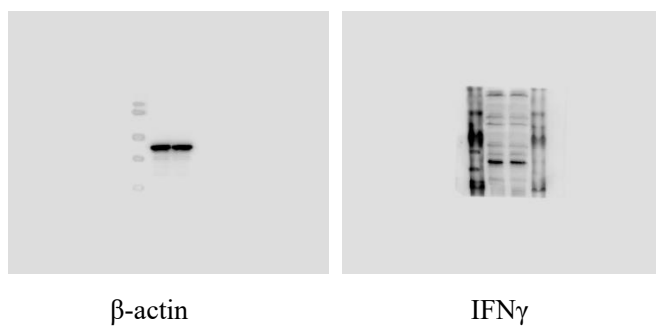

Figure 4H

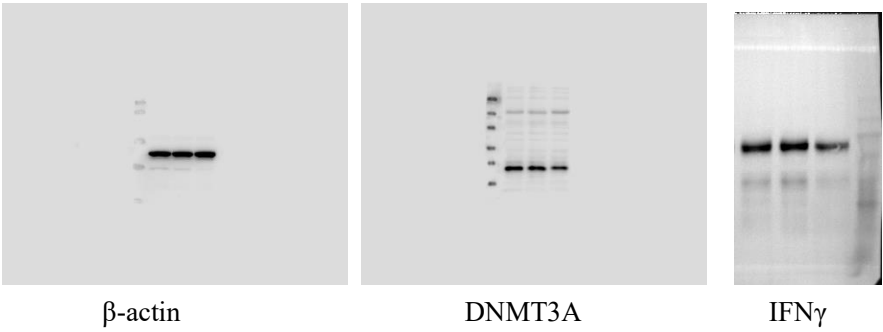

Figure 6E

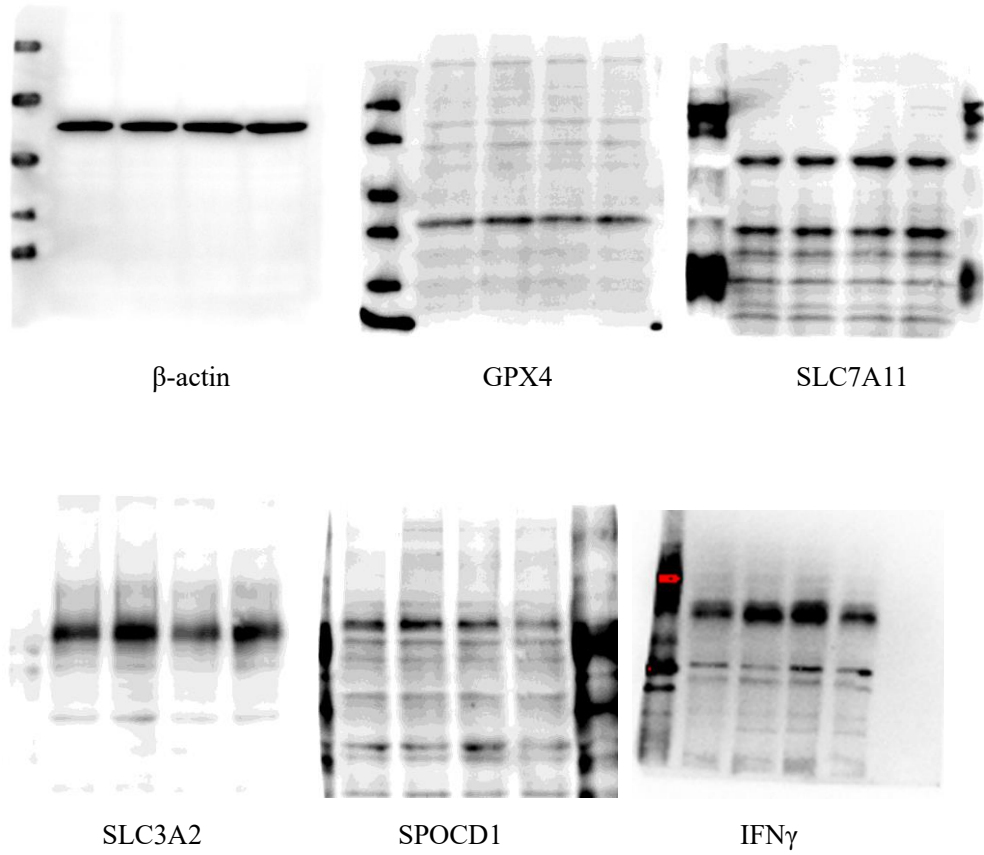

Supplement: Supplementary file 9 — Suppl. western blot [file 41420_2024_2257_MOESM9_ESM.pdf]
